# Understanding the Implications of Delaying Seasonal Influenza Vaccine Recommendations: An Industry Perspective

**DOI:** 10.3390/vaccines13090891

**Published:** 2025-08-22

**Authors:** Steven Rockman, Karen Laurie

**Affiliations:** 1 CSL Seqirus Ltd., Melbourne, VIC 3000, Australia; steve.rockman@seqirus.com; 2 Department of Immunology and Microbiology, The University of Melbourne, Parkville, VIC 3010, Australia

**Keywords:** seasonal influenza, influenza vaccine, timing of vaccine recommendations, vaccine, vaccine effectiveness, vaccine impact

## Abstract

Multiple studies have assessed the potential for improvement for genetic and antigenic match of influenza vaccines to circulating viruses by altering the timing of vaccine strain decisions. The advent of new technologies for vaccination has generated global discussion around moving the seasonal influenza strain recommendations closer to the start of the vaccination period. The window between influenza vaccine strain recommendations and the availability of vaccine supply for immunization comprises sequential processes required to produce vaccine components, reagents for manufacture and release, and regulatory approvals. This commentary examines one company’s perspective on requirements for enabling manufacture and release of seasonal influenza vaccine in more detail, describes preparations to reduce risk, and highlights the potential impact on vaccine supply for all platforms (egg, cell, mRNA) when strain decisions are issued closer to the desired vaccination timing.

## 1. Introduction

Each year, approximately 1.5 billion doses of seasonal influenza vaccine are produced, including trivalent influenza vaccines (TIV) containing A(H1N1), A(H3N2), and B/Victoria antigens and quadrivalent influenza vaccines (QIV), which include an additional B/Yamagata antigen. The vast majority of influenza vaccines (89%) are produced in embryonated chicken eggs, with the remaining 11% produced in either mammalian (Madin Darby canine kidney [MDCK]) or insect (baculovirus vector in expresSF + insect cells) cell lines [1]. Vaccines produced in eggs or MDCK cell culture may be either inactivated or live attenuated viruses, whereas those synthesized within insect cell lines comprise recombinant protein antigens. Based on the current capacity for seasonal influenza vaccine manufacture, 4.1 to 8.3 billion doses of vaccine are projected to be available within 12 months of an influenza pandemic [1].

Influenza A and B viruses are enveloped, negative-sense, single-stranded RNA viruses. The primary viral surface glycoproteins—hemagglutinin (HA) and neuraminidase (NA)—facilitate infection and transmission by mediating viral entry into (HA) and release from (NA) respiratory cells. HA and NA are the most antigenically variable proteins in the influenza virion and also the main targets for protective antibodies induced by influenza virus infection and vaccination [2]. Consequently, as they replicate, influenza viruses accumulate mutations in HA and NA. This evolutionary process is known as *antigenic drift* and is a key mechanism by which seasonal influenza viruses attempt to evade antibody responses to natural infections as well as seasonal influenza vaccines. It has been estimated that antigenic drift may reduce antigenic match between an influenza vaccine and circulating virus by 8% to 24%, and influenza vaccine effectiveness by 5% to 20% [3].

**Figure 1:**
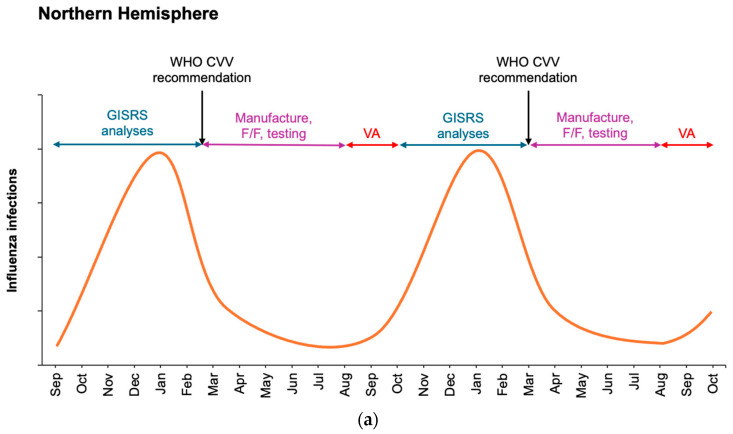

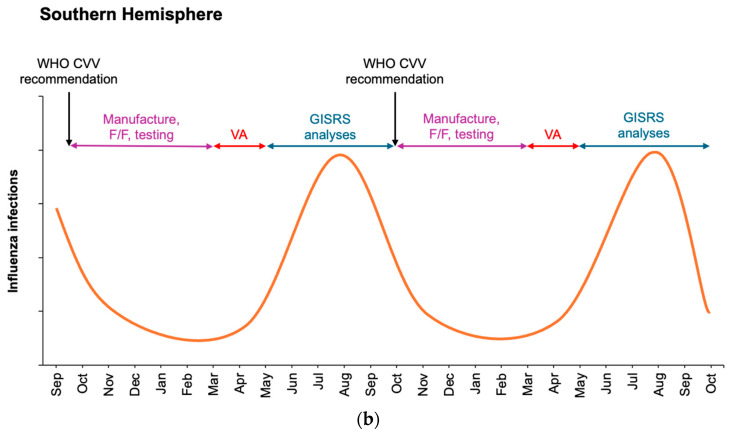
Schematic of influenza seasonality in the Northern (**a**) and Southern (**b**) Hemispheres detailing surveillance monitoring and assessment of circulating viruses by WHO GISRS laboratories during winter months and manufacture of influenza vaccines at the end of each influenza season in each hemisphere. Potential overlap is not depicted for clarity. Current WHO recommendations for seasonal influenza vaccine updates occur at the end of February for the Northern Hemisphere (**a**) and the end of September for the Southern Hemisphere (**b**). Vaccination administration (VA) ideally occurs immediately prior to the wintertime increase in influenza. F/F, fill/finish.

Consequently, seasonal influenza vaccines require ongoing updates of virus strains. Continuous surveillance of circulating influenza viruses is performed by laboratories of the World Health Organization Collaborating Centres for Reference and Research on Influenza (WHO CC) within the WHO Global Influenza Surveillance and Response System (GISRS). Antigenic match between circulating strains and vaccines is determined using serological analyses and genetic monitoring. If an antigenic mismatch is identified between circulating strains and current vaccines, new candidate virus vaccines (CVVs) are identified to improve subsequent vaccine match [4,5]. This process is performed annually for the Northern and Southern Hemispheres (NH and SH, respectively), since influenza infection rates peak in the temperate winter. Towards the end of each season in each hemisphere, the WHO collates and analyzes data and issues a recommendation for the composition of the seasonal influenza vaccine for the following winter season in that hemisphere (Figure 1) [6].

The important window of seasonal influenza vaccine availability is from the time of the recommendation made by the WHO to the delivery of sufficient doses to cover the forthcoming influenza season. With the egg- and cell culture-based influenza vaccine technologies currently in use, strain recommendations are made approximately 6 months in advance of vaccine availability to allow time for all the needed manufacturing steps, and 9 months in advance of the wintertime peak in influenza circulation [6]. Emergence of a shifted influenza virus, with consequence of an influenza pandemic, drives altered manufacturing responses [7,8].

Recently, there have been global discussions on the potential of vaccines manufactured on new and emerging platforms to shorten the time between the seasonal influenza vaccine recommendations and vaccine release [9,10,11]. Studies have assessed whether delaying the CVV strain announcement might improve the likelihood that selected strains would match viruses circulating during each influenza season, thus improving the performance of the vaccine (see discussion below). Some authors have also highlighted the potential advantages of co-administration of respiratory vaccines [9]. Understanding the process and challenges enabling timely vaccine delivery can inform future discussion on the timing of vaccine recommendations for seasonal influenza vaccines. This commentary details past challenges to vaccine delivery and collaborative strategies used to overcome those barriers. Newer vaccine platforms are also discussed in the context of currently licensed vaccines.

## 2. Timeline for Vaccine Manufacturing: From Candidate Vaccine Virus to Vaccine Administration

**Figure 2:**
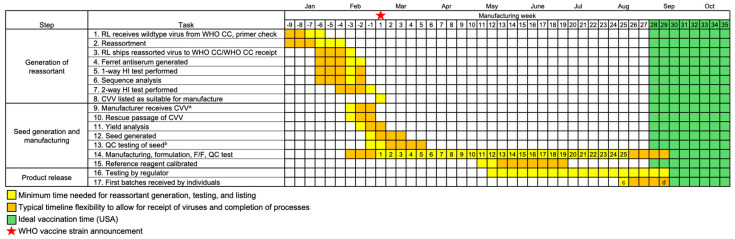
Steps and timeline of generation of a reassortant CVV and manufacture to first release of egg-based influenza vaccines for a typical NH season. Reassorting laboratories (RL) generate reassortant CVVs “at risk” (i.e., before vaccine strains are announced) in preparation for the WHO strain recommendation, which occurs at the end of February for NH seasons (star, week 1). Manufacturing, formulation of trivalent/quadrivalent vaccine, fill/finishing (F/F) in syringes, and quality control (QC) testing are grouped together for simplicity. Potency testing of product can only begin once antigen reference reagents are calibrated. Timing (*yellow, orange*) is from start of manufacturing until first vaccines are received by recipients based on UK/USA data [14,15,16], with week numbers indicating the minimum manufacturing weeks available (continued production/release not included for clarity). Ideal time to vaccinate (*green*) is as recommended in the USA [17]. ^a^ Reassorted CVVs can be shared with manufacturers once a virus has passed 1-way testing with the hemagglutination inhibition (HI) assay. ^b^ Sterility and yield assessments are performed. ^c^ Historical first influenza vaccine release in USA (2020–2021 season) [16]. ^d^ Historical first influenza vaccine release in UK (2023–2024 season) [14,15]. Vaccine schedules (e.g., US and UK) are locally determined.

All currently approved influenza vaccine manufacturing platforms rely on the identification of the most appropriate CVV to generate a vaccine using the whole virion in egg- and cell-based production or the appropriate hemagglutinin sequence in the case of recombinant influenza vaccines [12]. Both egg-based and cell-based technologies require a high-yield CVV, whereas recombinant-based technology requires robust baculovirus expression. These CVVs are generated by reassortment, in which a circulating wildtype influenza virus is mixed with a high-yielding virus under antibody pressure. Reassortant viruses may also be generated using individual gene transfection through reverse genetics rescue [13]. The progeny reassortant CVV expresses the surface antigens of the target circulating influenza strain and typically the internal genes of the high-yielding strain. WHO CCs provide circulating wildtype viruses to reassorting laboratories to generate reassortant CVVs and the sequences to recombinant manufacturers. These CVVs are then returned to the WHO CCs for antigenic and genetic testing to ensure match to the circulating wildtype strain (Figure 2, Tasks 1–3). Throughout the year, reassorting experiments and testing of reassortants may be performed “at risk”—that is, before the WHO announces the recommended strains for inclusion in the forthcoming influenza vaccine, which occurs at the end of February for the NH influenza season and at the end of September for the SH season. Prior to the strain recommendation meeting, WHO CCs collate information about the generation, testing status, and availability of reassortants. After the recommendation is announced, any CVV that is considered acceptable for inclusion in a vaccine in the forthcoming season is listed on the WHO website.

Notably, reassortment does not guarantee generation of a CVV with yield sufficient for manufacture of enough doses in the time between strain announcement (week 1) and vaccine availability (weeks 22–26). To help prevent generation of low-yield CVVs, different viruses from a single genetic clade are reassorted by reassorting laboratories. Each manufacturer therefore tests multiple available CVVs to determine the optimum candidate for their manufacturing process (Figure 2, Tasks 9–12).

**Figure 3:**
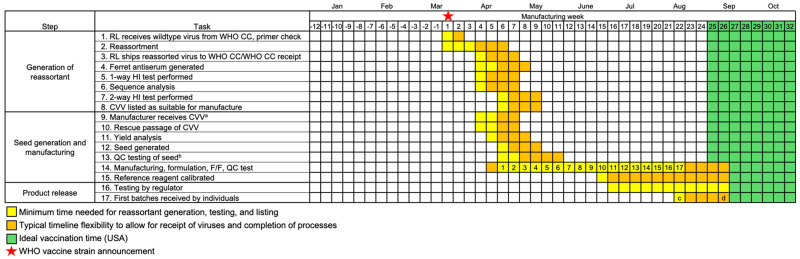
Timeline for vaccine production when the WHO recommendation is delayed by 4 weeks (indicated by star, week 1) due to a late-breaking strain owing to circulation patterns or the lack of a reassorted CVV at the time of the recommendation. Dates for CVV generation are based on generation of reassortant CVVs in September 2017 (IVR–186) and March 2019 (IVR–195). As in Figure 2, timing (*yellow, orange*) is from start of manufacturing until first vaccines are received by recipients based on UK/USA data [14,15,16], with week numbers indicating the minimum manufacturing weeks available. Ideal time to vaccinate (*green*) is as recommended in the USA [17]. ^a^ Reassorted CVVs can be shared with manufacturers once a virus has passed 1-way testing with the hemagglutination inhibition (HI) assay. ^b^ Sterility and yield assessments are performed. ^c^ Historical first influenza vaccine release in USA (2020–2021 season) [16]. ^d^ Historical first influenza vaccine release in UK (2023–2024 season) [14,15].

Once the WHO has issued the strain recommendations, the start of manufacturing will depend on whether there is a change in the recommended strain(s) and the availability of CVVs. The CVVs selected (or viral sequences) may either be (Scenario 1) currently used in manufacture (when there is no change to the recommendation for that strain), (Scenario 2) newly available for manufacture (the recommendation has changed, and CVVs have been generated and tested at risk), or (Scenario 3) not yet available (change in the recommendation and CVVs have not been generated or tested). For Scenario 1, reassortment is not required and manufacturing commences with viruses from the preceding hemispheric campaign. For Scenario 2, rescue passage, yield analysis, and seed generation typically take 3–4 weeks (Figure 2, Tasks 9–13) [18,19]. These steps may have already occurred prior to the WHO announcement, which permits manufacture to commence sooner than later. Scenario 3 might arise due to changes in circulation patterns leading to late-breaking identification of a strain or because a suitable reassorted CVV is not available. In such cases, Tasks 1–13 must be condensed into approximately 8 weeks (Figure 3).

## 3. Impact of Delaying Strain Announcement

What are the potential benefits of delaying the strain announcement for seasonal influenza vaccines? A thorough modeling study assessing virus circulation and antigenicity for past NH influenza seasons suggests that delaying selection could result in epidemiological and public health benefits due to improved antigenic match between the vaccine and circulating viruses [10]. However, a significant drawback to delaying the strain announcement to include emerging surveillance information is that the necessary reassorting work is unlikely to have been completed, nor will the reagents have been generated, thus jeopardizing timely, adequate vaccine supply to enable vaccination prior to the forthcoming season. In the modeling study, a benefit was shown for only one of nine NH seasons or 1 in 36 strains of a quadrivalent vaccine. In the single season where a benefit was observed, data indicative of an update were not available until 3–4 months after the current timing of the vaccine strain recommendation (the virus clade that dominated in the subsequent winter season was detected at <10% prevalence at the timing of the recommendation, increasing to >10% prevalence in July and 25% prevalence in August,). This would halve the time for vaccine preparation, testing, and delivery and potentially delay vaccine availability well past the ideal vaccination window [10].

Two recent examples of delayed availability of a CVV for vaccine manufacture provide further insights. First, in 2017, A/Singapore/INFIMH–16–0019/2016 was recommended for the SH 2018 A(H3N2) vaccine component. Although the reassortant CVV, NIB–104, was available, the yield did not meet our manufacturing requirements, and alternate reassorting commenced. The new reassortant, IVR–186, was generated and listed for use 7 weeks after the WHO recommendation. Second, in February 2019, the recommendation for the A(H3N2) component of the NH 2019–2020 vaccine was delayed by 4 weeks, from 22 February 2019, when the decision was originally scheduled, to 22 March 2019, when A(H3N2) A/Kansas/1/2017 was recommended, with the reassortant X–327 listed for use and another reassortant, IVR–195, listed pending two-way testing. In both examples from 2017 and 2019, manufacturing of one strain out of four was delayed by 5–7 weeks. Calibrated reference reagents, which are required for release of vaccine to the market, were available approximately 9 weeks after the CVVs were listed (Figure 3).

These examples demonstrate that revising the date of the WHO strain recommendation may have a substantial, negative impact on vaccine availability. Currently, NH strain selections are announced in late February, with the first doses delivered in August for the US and September for the UK [14,15,16], with an ideal vaccination time of September through October in the US and autumn through early winter in the UK (Figure 2) [17,20]. If epidemiological data were collected over an additional 4 weeks, the NH vaccine production window would be reduced from 25 weeks to 15–17 weeks (Figure 3). In the SH, where the strain announcement is in late September and doses are due early March, a campaign window of approximately 23 weeks may be reduced to 12–14 weeks. Decreasing the vaccine production time by 9–11 weeks may potentially reduce the number of doses available during the optimal distribution period prior to seasonal influenza peaks. In addition, reducing the production timeline eliminates any margin of error to compensate for manufacturing delays. Even in the standard timeline represented in Figure 2, timely delivery of vaccines becomes uncertain with a low-yield CVV, which necessitates more manufacturing runs to produce the required antigen yield. A low-yield CVV in the compressed scenario shown in Figure 3 would further reduce the number of available vaccine doses.

## 4. Impact of Newer Vaccine Technologies on Vaccine Timelines

All three currently utilized platforms—egg-based, cell-based, and recombinant—depend on the availability of CVVs (viruses or sequences) from WHO CCs and the manipulations and testing outlined in Figure 2. For egg-based influenza vaccines, egg supplies are secured in advance to enable manufacturing to proceed without delay.

Following acquisition of the cell-derived CVV, the timeframe for viral propagation in MDCK cells is generally similar to that of egg-based vaccine development.

Recombinant vaccine manufacture utilizes an insect cell system in which the HA gene from the cell-derived CVV is expressed by a baculovirus within an insect cell line. The resulting antigen, which is identical to the original HA sequence, is then purified and formulated as the recombinant vaccine [18,21]. The process to produce a baculovirus containing influenza antigen is similar to reassorting a virus [22,23], and the timely availability of this vaccine is also dependent upon an adequate yield of antigen produced in transduced cells.

What of mRNA vaccines? The COVID-19 pandemic accelerated the development and use of mRNA technology, enabling a new vaccine platform for the prevention of infectious disease. Like recombinant influenza vaccines, the sequence of the antigen gene for mRNA vaccines is ideally identical to the recommended strain, thus ensuring antigenic identity [24]. Preparative steps for the manufacture of an mRNA vaccine involves the generation of a candidate vaccine DNA template (which is amplified, purified, and linearized according to current good manufacturing practices) [25] before being transcribed and packaged as finished vaccine. Based on observations from updates to the COVID-19 vaccines in 2022 (addition of one variant to a bivalent vaccine) and 2023 (return to a monovalent vaccine with a single variant update), mRNA vaccines have become available 11–17 weeks after the recommendation made by the WHO Technical Advisory Group on COVID-19 Vaccine Composition (TAG-CO-VAC) [26,27,28,29,30,31]. Many activities for these COVID-19 vaccines were performed at risk, prior to the announcement of the variant to be used in the vaccine, including the preparation of constructs of circulating strains, preclinical evaluation, and some clinical testing [32]. Similarly to other methods of manufacture, if the decision for vaccine composition was delayed and the strain had yet to be identified, readiness to supply is estimated to occur at a range of 10–22 weeks [29,30,33]. Furthermore, like current methods, mRNA vaccines require potency testing with reagents—a critical step in the release of vaccine doses. All vaccines, including mRNA vaccines, require fill and finish into syringes and delivery to the vendors for vaccine distribution and administration. Completion of these tasks would likely translate into a timeframe for mRNA vaccine availability similar to the timelines for existing seasonal influenza vaccines.

## 5. Conclusions

In this commentary, we have analyzed the potential impacts of a delay in announcing a single component of the influenza vaccine. However, the cocirculation of A(H1N1), A(H3N2), and B/Victoria means that multiple component updates to a recommendation may occur. A common approach to optimizing the delivery of seasonal influenza vaccine is to manufacture at risk. If there was a delay in the vaccine composition announcement for the TIV to a later date, it would increase the risk of change in recommendations for all three vaccine components. A delayed announcement for all three influenza strains could have an even greater negative impact on vaccine delivery and population coverage than the scenarios outlined above. These risks exist for all types of vaccine platforms.

Various strategies to improve the effectiveness of influenza vaccines are available. Adjuvants can be used to improve the magnitude and breadth of the immune response, and higher antigen doses have been used to improve the magnitude of the immune response, particularly in older adults [34,35,36,37,38,39]. Both adjuvanted and higher dose vaccines are recommended for use in older adults [40,41,42]. In recent years, influenza surveillance has failed to detect B/Yamagata, leading to the removal of this strain from vaccine recommendations [43,44]. Alternative quadrivalent formulations containing an additional influenza A or B strain of the same subtype, or a double dose of one vaccine component, might also help maximize the antibody response to influenza vaccines.

To provide sufficient quantities of seasonal vaccines for the global population, all stakeholders should reach an understanding of the timeframes required for vaccine production for all platforms. This would help ensure timely supply of enough influenza vaccine doses to prevent infections and reduce the rate of influenza complications—the ultimate goal of influenza vaccines.

## Data Availability

No new data were created or analyzed in this study.

## Poster Presentation

This article is a revised and expanded version of a paper entitled “Delaying the Seasonal Influenza vaccine Strain Recommendation and the impact upon vaccine availability” by S Rockman and KL Laurie, which was presented as a poster at OPTIONS XII for the Control of Influenza 2024 Congress, held in Brisbane, Australia from 29 September to 2 October 2024 [45].
